# A Systematic Review of Randomized Controlled Trials on the Effectiveness of Computer-Tailored Physical Activity and Dietary Behavior Promotion Programs: an Update

**DOI:** 10.1007/s12160-012-9384-3

**Published:** 2012-07-06

**Authors:** Karen Broekhuizen, Willemieke Kroeze, Mireille NM van Poppel, Anke Oenema, Johannes Brug

**Affiliations:** 1 EMGO+ Institute for Health and Care Research, Amsterdam, Netherlands; 2 Faculty of Earth and Life Sciences, Vrije Universiteit Amsterdam, Amsterdam, The Netherlands; 3 Department of Health Promotion, School for Public Health and Primary Care (CAPHRI), Maastricht University, Maastricht, The Netherlands

**Keywords:** Computer tailoring, Physical activity, Dietary behavior, Primary prevention

## Abstract

**Background:**

A review update is necessary to document evidence regarding the effectiveness of computer-tailored physical activity and nutrition education.

**Purpose:**

The purpose of this study was to summarize the latest evidence on the effectiveness of computer-tailored physical activity and nutrition education, and to compare the results to the 2006 review.

**Methods:**

Databases were searched for randomized controlled trials evaluating computer-tailored physical activity and nutrition education aimed at primary prevention in adults, published from September 2004 through June 2011.

**Results:**

Compared to the findings in 2006, a larger proportion of studies found positive effects for computer-tailored programs compared to generic or no information, including those for physical activity promotion. Effect sizes were small and generally at short- or medium-term follow-up.

**Conclusions:**

The results of the 2006 review were confirmed and reinforced. Future interventions should focus on establishing larger effect sizes and sustained effects and include more generic health education control groups and objective measurements of dietary behavior.

**Electronic supplementary material:**

The online version of this article (doi:10.1007/s12160-012-9384-3) contains supplementary material, which is available to authorized users.

## Introduction

The potential impact of physical activity and healthy dietary habits on the prevention of a range of chronic conditions is substantial [1, 2]. Effective physical activity and dietary promotion interventions are needed. Successful intervention strategies and techniques to motivate and guide people to adopt healthy choices need to be identified. Over the last decades, computer tailoring has proven to be an innovative and promising health education technique [3–12]. A computer-tailored intervention mimics interpersonal counseling using a computerized process, but, unlike interpersonal counseling, it can be widely distributed through interactive media channels at a relatively low cost. Computer tailoring allows for individualized feedback and advice on personal behavior, personal motivation, outcome expectations, self-efficacy, social and physical environmental opportunities, and other behavioral determinants.

In recent years, a number of systematic reviews and meta-analyses have been published on the effectiveness of computer-tailored health education covering a range of behaviors [4, 5, 9, 10, 13, 14]. The effects of tailoring may, however, be behavior specific. It has been argued that computer tailoring may be especially promising for complex health behaviors, such as physical activity and dietary behaviors [15]. Examples of complex health behaviors are gaining increased awareness of personal behavioral patterns, comparing one's own behaviors with recommendations, and setting and monitoring progress toward behavior change goals. The first systematic review that explicitly focused on the effectiveness of computer-tailored health education on physical activity and dietary behaviors was published in 2006 and included intervention studies published up to September 2004 [3]. In concordance with other more narrative reviews on computer-tailored health education [15, 16], the authors concluded that computer tailoring was promising, especially for dietary behaviors, although the effect sizes were small. The authors made key recommendations for improving research on computer tailoring, i.e., using objective outcome measures instead of self-report or using generic health education comparison groups instead of or in addition to no-intervention control groups. The latter would allow more precise evaluation of the effects of tailoring health education interventions. Finally, it was concluded that longer follow-up was needed to assess the sustained effects in all studies.

Since many original studies have been published since 2004, a review update is needed to document evidence regarding the effectiveness of computer-tailored physical activity and nutrition education programs. Furthermore, responding to recommendations made in 2006, comparing effects and specific study and intervention characteristics over time, is additive to other systematic reviews and meta-analyses. This review update aims to: (1) review the evidence on computer-tailored physical activity and nutrition education from studies published since September 2004, (2) compare the evidence from this review update to that derived from the original review regarding intervention characteristics, study characteristics, and effects, and (3) provide updated recommendations for further research and practice.

## Methods

This paper reports on a second systematic review conducted using the study protocol of the original 2006 review. This protocol was based on guidelines extracted from the Cochrane Reviewers' Handbook [17].

### Search Strategy and Data Sources

For the original review, intervention studies published from 1965 to September 2004 were identified through a structured computerized search of PubMed, PsychInfo, and Web of Science. For this update, a nearly identical search was conducted from September 2004 to June 2011. The review differed from 2006 as we added the search engines' most recent thesaurus terms, resulting in the following search terms for nutrition: ((nutrition OR feeding OR food OR diet OR dietary OR intake OR nutritional status OR feeding behavi* OR food consumption) AND (education OR behavior OR behavio* OR education)) AND (tailored OR tailoring OR tailor* OR expert system) and for physical activity: (exercise OR motor activity OR sports OR leisure activities) OR (physical* AND active) OR (physical* AND activity) OR (physical* AND activities) OR exercis* OR walking OR cycling OR sport* OR leisure activit* AND (education OR behavior OR behavio* OR education) AND (tailored OR tailoring OR tailor* OR expert system). No limitations for age or study design were added.

### Selection of Studies

Just as in the original 2006 review, new studies had to examine a computer-tailored intervention aimed at promoting healthy physical activity or dietary behaviors for primary prevention of chronic diseases in apparently healthy adults. Evaluation studies that used a randomized controlled trial were included. Tailoring was defined by Kreuter as “the intention to reach one specific person, based on characteristics that are unique to that person, are related to the outcome of interest, and have been derived from an individual assessment” [18]. Interventions were considered to be computer tailored if the tailored advice was generated through a computerized process. Randomized controlled trials were included if: (1) published in a peer-reviewed scientific journal, 2) published in English, and 3) conducted in an adult sample (18+ years). Studies were excluded if the tailored intervention was part of a larger intervention program that made it impossible to isolate the effect of tailoring components from the other intervention components.

### Data Extraction

Detailed information was extracted only from new studies that met the aforementioned inclusion criteria. Two reviewers independently summarized the new studies for content and methods. The following intervention characteristics were extracted: theories used for intervention development, variables used to tailor the computer-tailored information, the “tool” that was used to provide individual feedback, frequency of tailored feedback, and additional health education activities. Extracted study characteristics were: the country where the study was conducted, size and source of the study population, eligibility criteria, intervention modes, and primary outcome measures. Results from single and multiple post-test measurements were extracted. The outcomes included all physical activity and dietary behavior measures. To interpret and compare results from the studies that used differing measures to assess physical activity and dietary outcomes, effect sizes (ESs) were calculated if significant effects were found (provided the data were available). The effect size, Cohen’s ES, was calculated by dividing the difference between two means at follow-up by their pooled standard deviation [19, 20]. Cutoff points for ESs were 0.2–0.5 for small ES, 0.5–0.8 for moderate ES, and >0.8 for large ES [21]. The findings were summarized per behavioral outcome (physical activity, fat intake, fruit and vegetable consumption, and other dietary behaviors) and separately for short- (<3 months), medium- (3–6 months), and long-term (>6 months) follow-up.

Apart from reporting the results found in the current review, we compared these with the results of the original 2006 review. In order to check whether recommendations from the original review were met, we compared intervention and study characteristics of the present review with the original one. Frequencies on the number of studies that found significant effects, as well as the number of studies that used objective outcome measures, various types of comparison groups (generic health education versus no-intervention control groups), and long-term follow-up, as well as delivery mode (printed versus electronically) are provided, linked to the original or current review.

## Results

### Study Selection

The initial cross-database search resulted in 2,590 publications. After eliminating duplicates, 1,562 remained. Titles and abstracts were reviewed for eligibility criteria, resulting in 141 publications that were fully considered. Fifty publications were finally included: 29 studies on physical activity and 34 on dietary behaviors, 21 on fat consumption, 18 on fruit and vegetable consumption, and 14 on other dietary topics. Other dietary topics included: energy/carbohydrate intake, the consumption of sugar, dairy, fiber, whole grain, and body fat, as well as weight and waist circumference. Thirteen studies in the current review evaluated interventions that targeted both physical activity and diet. Some publications reported on the characteristics and effects of one intervention using various follow-up measurements (e.g., short- and long-term effects) [22–26, 39], effects in a variety of study samples [27–30], effects on other types of outcomes (e.g., fruit intake and variety of fruit intake) [31], or the effects of various doses of the intervention (e.g., delivered at once or at multiple time points) [32, 33]. As a consequence, this review update reports on the characteristics and effects of 25 interventions targeted at physical activity, 27 interventions targeted at dietary behavior, and 10 interventions for both behaviors. Of the 27 interventions on dietary behavior, 17 were directed at fat reduction, 14 at increasing fruit and vegetable intake, and 12 at other dietary behaviors. The main reasons for exclusion were: the age of the study population was not in the required range, lack of randomized controlled trial design, no focus on primary prevention, absence of behavioral outcomes, or the computer tailoring was part of a multicomponent intervention that made it impossible to isolate the effect of tailoring.

### Intervention Characteristics

Characteristics of the interventions from studies in the current review are summarized in the Electronic Supplementary Material. Both physical activity and nutrition education interventions were predominantly guided by the Transtheoretical Model and Social Cognitive Theory. Most interventions (81 % of physical activity, 84 % of nutrition) provided tailored feedback on self-reported behavior. Two interventions (4 %) also provided feedback based on more objective data obtained from pedometers [34] or accelerometers [35]. Most interventions (92 % of physical activity, 68 % of nutrition) were tailored on presumed behavioral determinants such as intention, motivation, and stage of change, as well as self-efficacy and skills. Regarding nutrition education interventions, equal numbers of interventions provided print-delivered and electronically tailored feedback; however, the majority of physical activity interventions used electronic feedback formats (see also Table 1). Some interventions using electronic feedback had additional online discussion/message boards [36–38] (6 % of all interventions) or an e-buddy system (2 % of all interventions) [22, 38]. Electronic feedback was given on-screen (41 % of all interventions), by email reports (10 %), CD-ROM (4 %), or by mobile phone (2 %). Approximately one third of the interventions provided additional information such as booklets or information sheets. One intervention included weekly home visits [26, 39]. Less than half of the interventions provided tailored feedback more than once for dietary behaviors (48 %), and 65 % did so for physical activity.

**Table 1:** Study characteristics and effects of studies from the original (before 2004) and updated review (after 2004) compared

	Dietary behavior		Physical activity	
	Before 2004 (*N* = 26)	After 2004 (*N* = 34)	Before 2004 (*N* = 10)	After 2004 (*N* = 29)
	Reference number^a^	Reference number	Reference number^a^	Reference number
	*N* (%)	*N* (%)	*N* (%)	*N* (%)
Comparison of computer-tailored intervention with a no intervention control group	[33–35, 39, 42–44, 46–48, 50–56, 60]	[29–31, 34, 36, 53, 60, 65, 70, 71, 74, 78, 79, 82]	[33–35, 38]	[22, 23, 34, 36, 37, 60, 62–65, 74]
	18 (69 %)	14 (41 %)	4 (40 %)	11 (38 %)
Comparison of computer-tailored intervention with a generic health education control group^b^	[30–32, 40–42, 45, 54–56]	[24–26, 32, 33, 38, 39, 55, 56, 71–73, 75, 80, 81, 95]	[28–30, 32, 37, 38]	[24, 25, 32, 33, 35, 56, 59, 61, 66–69, 95–98]
	10 (38 %)	16 (47 %)	6 (60 %)	16 (55 %)
Objective measurements of effect indicators	[39, 50–52]	[24, 25, 34, 53, 56]		[24, 27, 28, 34, 35, 37, 66, 67, 69, 98]
	4 (15 %)	5 (15 %)	0 (0 %)	10 (34 %)
Inclusion of long-term (≥ 6 months) follow-up	[32, 33, 36, 43, 46]	[24–32, 34, 38, 39, 55, 56, 65, 70, 71, 75, 78–81, 95]	[28, 32–34, 36, 37]	[23, 27, 28, 32, 34, 35, 56, 61, 65, 67–69, 71, 95]
	7 (27 %)	23 (68 %)	6 (60 %)	14 (48 %)
Significant effects of computer-tailored interventions found	[30, 35, 39, 41, 43, 47, 49, 53, 56]	[24–34, 36, 38, 39, 55, 56, 60, 65, 70–75, 78–81]	[29, 35]	[22–25, 32–34, 36, 37, 54, 59, 60, 62–67, 74]
	9 (35 %)	28 (82 %)	2 (20 %)	19 (66 %)
Printed intervention materials	[30–34, 40–46, 48–50, 53, 54, 56]	[24, 26, 28–30, 32, 33, 39, 53, 73, 75, 77–79, 81, 95]	[28–34, 37, 38]	[22, 23, 27, 28, 32, 33, 64, 67, 68, 95]
	18 (69 %)	15 (44 %)	9 (90 %)	10 (34 %)
Electronic intervention materials	[35, 36, 39, 44, 47, 51, 52, 55, 60]	[34, 36, 38, 55, 56, 60, 70–72, 74, 80, 82]	[35, 36]	[24, 25, 34–38, 54, 56, 60–63, 66, 69, 96–98]
	9 (35 %)	12 (35 %)	2 (20 %)	18 (62 %)

*N* number of studies

^a^Reference numbers of studies < 2004 are derived from the original review [3]. Reference numbers of studies after > refer to references used in this review

^b^In some studies, a no-intervention and generic health education control groups were both included

### Study Characteristics

The characteristics and effects for studies in the current review are shown in the Appendix. The majority of studies were conducted in the USA, followed by the Netherlands and Belgium, the UK, and several other countries.

Studies in the USA predominantly assessed physical activity with the validated 7-day Physical Activity Recall [40–43]; this was the most commonly used tool. The next most common tool was the validated Short Questionnaire Assessing Health-Enhancing Physical Activity (SQUASH) [44] predominantly used by Dutch researchers. The International Physical Activity Questionnaire (IPAQ) [45, 46] was the third most commonly used assessment tool. Six studies (21 %) included objective assessments of physical activity, i.e., pedometer, actigraph, or accelerometer. Five studies (17 %) measured aerobic fitness by either a (1 mile) walking test [47, 48], the Chester step test [49], or the submaximal exercise treadmill test [50].

Fat reduction was most often assessed using food frequency questionnaires. In the USA, the Block questionnaire was used most frequently [51] and in the Netherlands, a questionnaire developed by Van Assema et al. [52]. Two studies obtained data from either an electronic scanner [53] or shopping receipts [34] in a supermarket setting. Data on fruit and vegetable consumption were obtained from questionnaires (the Block questionnaire in the majority of studies); one study also used shopping receipts [34]. Studies that included measures of weight or BMI either used self-report [38, 54] or measured [24, 27, 28, 34, 55, 56]. Fiber, grain, energy, or added sugar intakes were assessed by food frequency questionnaires [57, 58].

### Effects on Physical Activity (Section A, Appendix)

Of the 29 studies on physical activity, 20 (69 %) showed significant differences in favor of the computer-tailored intervention. Five studies looked at short-term effects [36, 37, 59–61], of which four found significant effects for the tailored intervention [36, 37, 59, 60] with small effect sizes, compared to no intervention. In one study, this applied to participants who did not comply to the physical activity guidelines at baseline [60]. Of the 17 studies with medium-term follow-up periods, 12 found significant effects with small effect sizes: six compared to no intervention [22, 36, 62–65], five compared to generic health education [24, 32, 33, 66, 67], and one compared to a health risk assessment [67]. Studies that investigated two computer-tailoring techniques [22, 54, 63, 67] found significant effects for both tailoring conditions. Six of the 13 studies with long-term follow-up found significant effects of the tailored intervention [23, 25, 32, 34, 65, 67]. Effect sizes were small except for one study that reported medium effect size for one of the two computer-tailored interventions investigated [67]. Of the eight studies that assessed effects at various follow-up periods, four studies reported no effects at either short, medium, or long term [35, 61, 68, 69]; six studies reported sustained effects over time[22, 23, 25, 34, 36, 65, 67], and one study reported no effect at short term but a significant effect at medium term [62].

### Effects on Fat Consumption (Section B, Appendix)

Of the 21 studies on fat consumption, 17 (81 %) showed significant differences in favor of the computer-tailored intervention. Six studies tested short-term effects and reported significant effects of tailoring compared to no intervention [36, 60, 70, 71], or generic health education [72, 73] with small effect sizes. Two of those studies (also) targeted an at-risk population [60, 72]. At medium term, all eight studies found significant effects compared to no intervention [36, 70, 74], or generic health education [33, 72–75]. One of those studies targeted a low-income ethnically diverse population [76], and a second study also found a significant effect among risk consumers (i.e., people with fat intake levels higher than recommended at baseline) [72]. Ten studies tested the long-term effects of an intervention, and five found significant effects for tailoring compared to no intervention [29, 30, 70] or generic health education [24, 32] with small effect sizes. Two of the ten studies (also) targeted high-risk populations [29, 30], and another study targeted women aged 50–69 years [24]. Multiple measurements in time were reported for seven studies, of which five studies reported sustained significant effects [25, 36, 70, 72, 73], one study reported a significant effect at short term [26] that was not sustained in the long term [39], and one study reported no effects at both medium- and long-term time periods [77].

### Effects on Fruit and Vegetable Consumption (Section C, Appendix)

Of the 18 studies on fruit and vegetable consumption, 15 (83 %) showed significant differences in favor of the computer-tailored intervention. Two of these studies measured the short-term effects of a computer-tailored intervention, and both found significant effects compared to no intervention [36, 71] with small effect sizes in a general population. Six studies measured medium-term effects, of which five found significant effects compared to no intervention [36, 65, 78] or generic health education [33, 75] with small effect sizes. One study investigated the effects of two intervention conditions (either delivered in one or four installments) compared to generic health education and measured the effects of retailored feedback [75]. The latter measured the effect of retailored feedback provided in four installments. Eight of the 12 studies that tested the long-term effects of an intervention found significant effects for tailoring interventions compared to no intervention [31, 34, 65, 79] or generic health education [24, 32, 80, 81]. The eight studies found small effect sizes, except for one that had targeted church members, which found a large effect size over the long term [31]. Two studies with effective long-term interventions targeted populations who were over 50 years of age [24, 56]. Heimendinger and colleagues found a significant effect of (re)tailored advice when spread across four booklets, as opposed to no effect when the advice was delivered in a single booklet [81]. Nine studies reported multiple measurements in time, and seven of these reported sustained effects [25, 32, 34, 36, 65, 75, 78]. One of the nine studies reported no medium-term effect but a significant long-term effect [79], and one study reported no medium- or long-term effect [77].

### Effects on Other Diet-Related Behaviors (Section D, Appendix)

Of the 14 studies on other dietary behaviors, 8 (57 %) showed significant differences in favor of the computer-tailored intervention. Four interventions for weight loss found significant effects including: one short, medium, and long term [28]; one medium and long term [38]; and two long term only [34, 55]. Effect sizes were small [34, 55], medium [28], or large [38]. Of the three interventions on energy intake, one reported a significant short- and medium-term effect [72]. The corresponding effect size was small for the general study population and medium among risk consumers in the short term. In addition, at medium term, only the effect of print-based advice (as opposed to delivery through CD-ROM) was of significance in the general population with a small effect size. Both studies considering fiber consumption found significant short-, medium- [70], and long-term effects [34] with small effect sizes. The intervention on grain intake showed no significant effect, nor did an intervention aimed at reducing added sugar. No significant effect was observed for the intervention to change dairy consumption [82].

### A Comparison Between the Present Update and the Original 2006 Review

The present review included 50 publications over just under 7 years, while the original review in 2006 included 30 publications over 13 years, showing an apparent increase in studies on physical activity and tailored nutrition education. This increase was most obvious for physical activity (29 studies in the present review, 11 in the original review).

Since 2004, the number of computer-tailored interventions electronically delivered has increased, particularly in physical activity studies (see Table 1). New delivery modes, such as mobile phone and CD-ROM, were introduced since 2004. Similar to the original review, in the majority of studies included in the present update, a no-intervention control group was included without a generic health education comparison group. Most studies continue to lack objective assessments of effects of nutrition interventions, but physical activity intervention studies often used objective assessments for behavior changes. As recommended in the original 2006 review, more nutrition intervention studies included long-term follow-up.

In this update, the majority of studies reported significant effects of computer tailoring, both for dietary and physical activity behavior (the largest increase). However, effects sizes remained small in general for dietary as well as physical activity behavior.

## Discussion

The present review update confirms and further strengthens the evidence that computer-tailored physical activity and nutrition education is likely to be effective [4, 5, 9, 10, 13, 14], although effect sizes related to tailored physical activity and nutrition education interventions are likely to be small. The evidence for long-term effects of computer tailoring remains inconclusive.

The present review is an update of a 2006 review of the literature published up to September 2004. A number of differences in the results of the original and updated review are noteworthy. First, both for physical activity and dietary behavior, the number of published studies has increased substantially. In addition, a larger proportion of published studies reported favorable effects of tailored interventions in the update period than in the original review. Evidence on the efficacy of computer-tailored education is now also apparent for physical activity promotion. Second, the use of objective outcome measurement instruments increased in studies on physical activity education, but not for nutrition education studies. Third, overall, there was no increase in comparisons of interventions with generic health education since 2004. Fourth, remarkably more studies with long-term follow-up were performed in the past years, particularly on nutrition education. Finally, the electronic delivery of feedback increased, particularly in studies on physical activity promotion; discussion boards/forums were frequently added to interventions.

The observed differences over time for the use of objective outcome measurements and various types of control groups, follow-up periods, and delivery modes require more attention. Since 2004, a larger number of objective measures have been included in tailoring studies, especially regarding physical activity education. In this field, accelerometers and pedometers have grown in popularity, due to increased usability and feasibility [83]. In the field of nutrition, no such development was seen. The objective measurement of dietary intake can be achieved by monitoring biologic dietary indicators, such as serum cholesterol and serum carotenoids [84]. However, the assessment of biologic indicators is relatively expensive, and these indicators are subject to genetic differences. Alternatively, two studies used shopping receipts and electronic shop scanners as objective indicators of food purchases [34, 53]. In addition, anthropometrics and waist circumference were the most frequent objective indicators.

The fact that the evidence in favor of computer-tailored physical activity and nutrition education is now stronger than based on the studies published up to 2004 is promising and important. However, the strongest evidence comes from studies that compared tailored interventions to no-intervention control groups. Thus, these studies could not assess the effects of tailoring compared to non-tailored interventions. Significant effects were most often found in studies with a no-intervention control group. These findings do not differ from the results of the original review or other comparable reviews [3, 6–8, 13]. Therefore, the evidence is stronger for a comparison between tailored interventions and with no intervention than with generic health education. However, this is probably because of the larger number of studies that included a no-intervention control group. If generic health education control groups were included in a study, the evidence was quite consistently in favor of tailoring. If this review had been restricted only to comparisons between tailored interventions with generic health education comparison groups, it would have focused specifically on the additional effects of tailoring in health education. Nevertheless, we believe that the comparison with no-intervention control conditions is also important, because it shows that tailored interventions are likely to be effective—because of the tailoring or other factors—and that is important information for health education practice. In addition, further exploration of the effectiveness of computer-tailored interventions compared to other control conditions, such as theory-based or personalized interventions, would be valuable to verify whether individually tailored education is better than theory-based and/or personalized education.

For physical activity and nutrition interventions to have an effect on health, the effects should be sustained over long periods of time [76]. The present review update shows that since 2004, more studies with long-term follow-up (>6 months) have been published. However, the positive effects of these studies were generally observed at short- and medium-term follow-up. Lack of long-term effects of health education interventions has been reported before. In a meta-analysis of computer-tailored interventions, Krebs and colleagues also found a significant trend of decreasing effect size when follow-up time increased [4]. Some evidence suggests that “dynamic tailoring” with more tailored feedback moments throughout a long intervention period may improve effects beyond the short term. The present updated review further shows that iterative feedback and tools supporting self-regulatory skills (e.g., goal setting activities, self-monitoring tools, skills building activities, email reminders, booster sessions, and interactive activities) are ways to realize such repeated tailoring [4, 5, 15, 85].

Not only has the number of electronically delivered interventions grown since 2004, but evidence for effectiveness has too. Before 2004, only a third of these “second-generation” dietary interventions were effective, compared to 60 % after 2004. For effective promotion of physical activity, the likelihood of effect appears not to be dependent on delivery mode. Furthermore, mobile phones were a delivery mode that was not yet available in the studies in the original 2006 review. A study by Haapala et al. indicates that mobile phone delivery can be an effective method for supporting weight loss. By allowing for two-way communication and showing a log-on frequency that is twice the rate of other web-based programs [86, 87], mobile phones have potential for the future. Because of these advantages and given the massive increase of the use of smartphones worldwide, mobile technologies will and probably should be used more often to promote lifestyle changes [88].

Overall, studies published since 2004 appear to have partially taken into account the recommendations for further research in the original review. Although more objective outcome measurement instruments were used in studies published after 2004, this was restricted to interventions on physical activity. Further, despite the increased number of studies, the proportion of comparisons with generic health education has not increased since 2004. Long follow-ups have been included more frequently in more recent studies, but only in nutrition interventions. Comparisons with generic health education, instead of no-intervention control groups, are most important because they provide information on the effects of tailoring. Therefore, we repeat and strongly advocate the recommendation to study tailoring as compared to other intervention methods, such as generic health education. Long-term follow-up should remain a priority, as well as the inclusion of objective outcome measures including their use in nutrition intervention research.

This review update has limitations. We used the same review protocol as was applied in the original 2006 review. Therefore, potential limitations such as the non-blinding of reviewers to authorship or the journal of the reviewed publications also applied to the present review. A lack of unequivocal scientific evidence that blinding is essential to obtain valid review results was already discussed in the original 2006 review [3, 89, 90]. In addition, a new independent reviewer assessed eligibility of the studies for the present update, which could have led to some differences in decisions and interpretations. Previous research has shown that updating a review can affect both the direction and the precision of the outcome [91, 92]. Yet, two reviewers who were involved in the reviewing process of the original 2006 review were also part of the present update team. No risk of bias and/or quality assessment evaluations were performed for either the original and updated review, although the use of such tools has been recommended for systematic reviews [17]. Fortunately, because only randomized controlled trials were included, the variety in methodological quality was small. Nevertheless, the methodological quality of the studies included in this review could have had an impact on estimates of effects, which might have affected the validity of the conclusions. Finally, as with any review of published literature, the present update may have been affected by publication bias that may have caused an overestimation of the positive findings.

Notwithstanding these potential limitations, this review importantly updates the systematic overview of developments and evidence regarding computer-tailored physical activity and nutrition education over the past years. Furthermore, this review update provides the most recent overview of the content and effects of computer-tailored interventions in the field of physical activity and nutrition. Reviews of the literature need to be updated regularly in order to provide up-to-date overviews of the evidence base to inform health promotion practice and to provide new recommendations for research to further strengthen the evidence base. This comparison is strengthened by our use of comparable reviewing methods at two time points, 2006 and 2011, giving us the opportunity to compare effects, intervention, and study characteristics over time. Such updating of reviews using a similar methodology is advocated and common practice in review consortia such as the Cochrane collaboration.

On the whole, from this updated review, it can be concluded that the evidence on computer-tailored interventions for the promotion of physical activity and dietary change has become stronger and now is also convincing for physical activity promotion. However, this effect particularly accounted for studies with no-intervention control groups, effect sizes were generally small, and the evidence is generally restricted to rather short-term effects, i.e., up to 3 months follow-up. Further, it remains unclear whether the effect of tailored interventions is caused by tailoring as such or by the fact that tailored interventions are more likely to be carefully designed and based on behavioral theory. Previously formulated recommendations regarding the use of objective outcome measurements, generic HE control groups, and long-term follow-up periods for the development of computer-tailored interventions were only partially met. Based on the present review, the use of computer-tailored interventions in physical activity and healthy nutrition promotion can be advocated, but future interventions should especially focus on: (1) establishing larger effect sizes and sustained effects, (2) using more objective measurements in studies on dietary behavior, (3) using more generic HE control groups and especially control groups in which the generic health education is also carefully designed and theory-based in order to distinguish the effect of tailoring from the effects of theory-based intervention development, and (4) including more long-term follow-up measurements. Future research should also focus on why and how computer-tailored physical activity and nutrition interventions are effective, by conducting mediation analyses [23, 93], and supporting large-scale dissemination of such interventions [94].

### Electronic Supplementary Material: Intervention Characteristics

Below is the link to the electronic supplementary material.

ESM 1 (DOCX 53 kb)

## Appendix

**Table 2:** Study characteristics and effects found in the studies included in the review

First author(s)^a^ [reference number]	Country	Study population [*N*]	Intervention modes^b^	Validated questionnaire	Outcome measurement instruments	Outcome measurement units	Results^c^ and effect size^d^ at short (ST), medium (MT), or long term (LT)^e^
A. Physical activity							
Adachi, 2007 [28]	Japan	Overweight Japanese women [205] recruited from the general population (Adachi, 2007)	**C** Self-help booklet	?	15-item	Self-rated physical activities (points 1 (bad)–3 (good)	**LT** No significant effects
Tanaka, 2010 [27]		Overweight Japanese men [51] recruited from the general population (Tanaka, 2010)	**EXP1** C + self-monitoring of weight and walking		Pedometer	Daily walking steps	
			**EXP2** CT advice				
			**EXP3** ^f^ CT advice + self-monitoring of weight and walking				
Carroll, 2010 [96]	USA	Inactive participants [394] recruited through primary care providers	**C** Generic HE	Yes	7-Day PA	Leisure-time PA (min/week)	**MT** No significant effects
			**EXP1** CT advice		Recall	Non-leisure-time PA (min/week)	
Dunton, 2008 [62]	USA	Women [156] (21–65) recruited from the general population	**C** No intervention	Yes	Standardized activity inventory	MVPA (min/week)	**ST** No significant effects
			**EXP1** CT advice			Walking (min/week)	**MT** Significant effect on MVPA
							ES: 0.24
							**MT** Significant effect on walking
							ES: 0.21
Hageman, 2005 [66]	USA	Women [31] (50–69 years) recruited through newspaper advertisement	**C** Generic HE	Yes	Modified 7-day physical activity recall	MVPA (min/week) calories expended daily	**MT** Significant effect on VO2 max
			**EXP1** CT advice		Fitness walking test	Aerobic fitness (VO2 max in ml/kg/min), flexibility (cm)	ES: 0.42
					Sit-and-reach test		
Hurling, 2007 [37]	UK	Participants [77] (30–55 years) recruited through market research recruitment agency	**C** No intervention	Yes	IPAQ	Overall PA (MET min/week)	**ST** Significant effect on leisure-time PA
			**EXP1** CT advice		Accelerometer	Leisure-time PA (MET min/week)	Accelerometer data
						Overall sitting time (h/week)	Significant effect on MPA (3–6 MET range)
						Weekday sitting time (h/week)	ES: N/A
						Weekend sitting time (h/week)	
Jacobs, 2004 [95]	USA	Women [511] (50–64) recruited from nutrition and PA program (WISEWOMAN)	**C** Generic HE	?	31-item PAA questionnaire	Score from 31-item scale: not very active (0)–very active (42)	**LT** No significant effect on PA score
			**EXP1** CT advice				
Marcus, 2007 [67]	USA	Sedentary participants [239] (18–65) recruited from the general population	**C** Generic HE	Yes	7-Day physical activity recall		
			**EXP1** CT advice (print-based)		Actigraph		
			**EXP2** CT advice (telephone-based)		Submaximal exercise threadmill test	MPA/VPA (min/week)	**MT** Significant effect on PA in EXP2 compared to C
						Aerobic fitness (VO2max in ml/kg/min)	ES: 0.46
							**MT** Significant effect on PA in EXP1 compared to C
							ES: 0.39
							**MT** No significant difference between EXP1 and EXP2
							**LT** Significant effect on PA in EXP2 compared to C
							ES: N/A
							**LT** No significant effect on PA in EXP1 compared to C
							**LT** No significant difference between EXP1 and EXP2
Marcus, 2007 [69]	USA	Sedentary participants [249] (18+) from the general population	**C** Generic HE	Yes	7-Day physical activity recall	MPA/VPA (min/week)	**MT/LT** No significant effect on MVPA
			**EXP1** CT advice (internet)		Submaximal exercise treadmill test	Aerobic fitness (VO2max in ml/kg/min)	
			**EXP2** CT advice (print-based)				
Napolitano, 2006 [68]	USA	Sedentary women [280] recruited from the general population	**C1** Generic HE	Yes	7-Day physical activity recall	MPA/VPA (min/week)	**MT/LT** No significant effect on MVPA
			**C2** Self-help booklet				
			**EXP2** CT advice				
Oenema, 2008 [60]	The Netherlands	Participants [2,159] (>30) recruited from online research panel	**C** No intervention	Yes	Short version of IPAQ	Self-rated PA level (scale from −2 to +2)	**ST** Significant effect on % compliant to PA guideline in at-risk group (those who did not comply with the PA guidelines at baseline)
			**EXP1** CT advice			% compliant to PA guideline (moderate intensity PA for at least 30 min/day in at least 5 days/week)	ES: 0.16
Pekmezi, 2009 [97]	USA	Sedentary Latinas [93] (18–65) recruited from the general population	**C** Generic HE	Yes	7-Day physical activity recall	MPA/VPA (min/week)	**MT** No significant effect on MVPA
			**EXP1** CT advice				
Prochaska, 2008 [54]	USA	Participants [1400] at risk for at least one risk behavior (exercise, stress, BMI >25 kg/m^2^ and smoking) recruited from a major medical university	**C** Health risk assessment	Yes	Self-reported level of exercise	% exercising moderately 30 min/day for at least 5 days/week	**MT** Significant effect on % exercising moderately 30 min/day for at least 5 days/week in EXP1 and EXP2 compared to C
			**EXP1** C + coaching				ES: N/A
			**EXP2** C + transtheoretic model-based feedback				
Quintiliani, 2010 [59]	USA	Female college students [408] recruited from universities/colleges	**C** Generic HE	Yes	US Behavioral Risk Factor Surveillance Survey	MVPA (min/week)	**ST** Significant effect on VPA in EXP2 compared to C
			**EXP1** CT advice (topic by choice)			VPA (min/week)	ES: 0.41
			**EXP2** CT advice (topic by expert)				
Slootmaker, 2009 [35]	The Netherlands	Participants [102] (20–40 years) recruited from worksites	**C** Generic HE	?	AQuAA[99]	LPA/MPA/VPA (MET min/week)	**MT/LT** No significant effects
			**EXP1** CT advice		Chester Step Test	Aerobic fitness (VO2max in ml/kg/min)	
Smeets, 2007 [33]	The Netherlands	Participants [2,827] (18–65) recruited from companies and the general population	**C** Generic HE	Yes	SQUASH	Action moments/week	**MT** Significant effect on PA of EXP1 compared to C
De Vries, 2008 [32]			**EXP1** CT advice (once delivered in 3 months (Smeets et al.))			% compliant to PA guideline (moderate intensity PA for at least 30 min/day in at least 5 days/week)	ES: 0.12
			**EXP2** CT advice (3 times delivered in 9 months (De Vries et al.))				**LT** Significant effect on PA and % compliance to PA guideline of EXP2 compared to C
							ES: 0.15
							ES: 0.14
Smeets, 2008 [64]	The Netherlands	Participants [487] (18–65 year) recruited from the general population	**C** No intervention	Yes	SQUASH	Total PA (MET min/week)	**MT** Significant effect on transport related PA and total PA among motivated participants
			**EXP1** CT advice			Transport related PA (MET min/week)	ES: 0.48
						Leisure-time related PA (MET min/week)	ES: 0.49
						Sports related PA (MET min/week)	
Spittaels, 2007 [63]	Belgium	Participants [434] (20–55 year) recruited through parents and staff of primary/secondary schools	**C** No intervention	**Yes**	IPAQ	Total MVPA (min/week)	**MT** Significant effect on transportation PA, leisure-time PA and weekday sitting time in EXP1 and EXP2 compared to C
			**EXP1** CT advice			Transportation PA (min/week)	*EXP2 compared to C*
			**EXP2** CT advice + repeated feedback			Household PA (min/week)	ES (transportation PA): 0.21
						Leisure-time PA (min/week)	ES (leisure-time PA): 0.52
						Job-related PA (min/week) weekday sitting time (min/day)	ES (weekday sitting time): 1.58
						Weekend sitting time (min/day)	*EXP1 compared to C*
							ES (transportation PA): 0.18
							ES (leisure-time PA): 0.40
							ES (weekday sitting time): 1.62
Spittaels, 2007 [98]	Belgium	Participants [526] (25–55 year) recruited from worksites	**C** Generic HE	**Yes**	IPAQ	Total PA (min/week)	**MT** No significant effects in EXP1 or EXP2 compared to C
			**EXP1** CT advice		Accelerometer	MVPA (min/week)	
			**EXP2** CT advice + stage-of-change based emails			30 min of PA on most days (%)	
Sternfeld, 2009 [36]	USA	Participants [787] recruited from administration offices of a large healthcare organization	**C** No intervention	Yes	Physical Activity Questionnaire adapted from Cross-Cultural Activity Patterns Questionnaire	Total PA (MET min/week)	**ST** Significant effect on MPA, VPA, walking, and sedentary behavior
						**EXP1** CT advice	**MT** Significant effect on MPA, walking, and sedentary behavior
						MPA (min/week)	**ST** Significant effect on MPA, VPA, walking and sedentary behavior among those who chose the PA path of the intervention
						VPA (min/week)	ES: N/A
						Walking (min/week	
						Sedentary behavior (min/week)	
Van Keulen, 2011 [65]	The Netherlands	Participants [1,629] (45–70) recruited from general practices	**C1** No intervention	Yes	28-item modified Community Health Activities Model Program for Seniors	PA (hours/week)	**MT** Significant effect of EXP1 compared to C1
			**C2** Coaching				ES: 0.20
			**C3** C2 + EXP1				**LT** (~11 months) Significant effect of EXP1 compared to C1 and C3
			**EXP1** TC advice				ES (EXP1-C1): 0.32
							ES (EXP1-C3): 0.15
							**LT** (~18 months) no significant effects
Van Stralen, 2009 [22]	The Netherlands	Participants [1971] (>50 years) recruited from Regional Municipal Health Councils	**C** No intervention	Yes	1-item from SQUASH	Self-rated PA (total weekly days of MPA)	**MT** (3 months) Significant effect on self-rated PA in EXP1 and EXP2 compared to C
Van Stralen, 2011 [23]			**EXP1** CT advice (psychosocial)			Self-rated compliance with PA guidelines (% of participants that show compliance with guidelines)	ES: 0.20
			**EXP2** CT advice (psychosocial + environmental)				ES: 0.20
							**MT** (3 months) Significant effect on PA initiation among insufficiently active participants in EXP1 and EXP2 compared to C
							ES: 0.26
							ES: 0.21
							**MT** (6 months) Significant effect on self-rated PA in EXP1 and EXP2 compared to C
							ES: 0.30
							ES: 0.35
							**MT** (6 months) Significant effect on PA initiation among insufficiently active participants in EXP1 and EXP2 compared to C
							ES: 0.32
							ES: 0.27
							**MT** (6 months) Significant effect on PA maintenance among sufficiently active participants in EXP 1 and EXP 2 compared to C
							ES: 0.33
							ES: 0.34
							**LT** (12 months) Significant effect on self-rated PA in EXP1 and EXP2 compared to C
							ES: 0.18 (for both EXP1 and EXP2)
Walker, 2009 [24]	USA	Women [225] (50–69) recruited from the general population	**C** Generic HE **EXP1** CT advice	Yes	Modified 7-day Physical Activity Recall	MVPA (min/day)	**MT** Significant effect on lower body muscular strength
Walker, 2010 [25]							ES: −0.36
					1 mile walk test Modified sit-and-reach test	Kilocalories expended per kilogram/day	**LT** (12 months) Significant effect on lower body muscular strength
					Repeated timed chair stands	Time engaged in strengthening and stretching exercise (min/week)	ES: −0.41
						Aerobic fitness (VO2max in ml/kg/min)	**LT** (18 months) Significant effect on lower body muscular strength
						Lower body muscular strength (timed chair stands in s)	ES: −0.51
Wanner, 2009 [61]	Switzerland	Participants [1,531] recruited from the general population	**C** Generic HE **EXP1** CT advice	?	4-item derived from official PA monitoring in Swiss population Accelerometer	MPA/VPA (min/week)	**ST/LT** No significant effect on MPA and VPA
Werkman, 2010 [56]	The Netherlands	Recent retirees [415] (55–65) recruited from pre-retirement workshops	**C** Generic HE **EXP1** CT advice	Yes	Dutch version of the PA Scale for the Elderly (PASE) [96]	Daily routine PA (min/week)	**LT** No significant effect (12 and 24 months) on daily routine PA, recreation/sports PA, Σ household activities (0–6) and PASE-score
						Recreation/sports PA (min/week)	
						Σ household activities (0–6) PASE-score (0–400)	
Winett, 2007 [34]	USA	Participants [1071] recruited from churches	**C** No intervention	?	Pedometer	Daily step counts	**LT (7 and 16 months)** Significant effect on PA in EXP2 compared to C
			**EXP1** CT advice				ES (7 months): 0.23
			**EXP2** CT advice + church support				ES (16 months): 0.27
B. Fat consumption							
Blair Irvine, 2004 [71]	USA	Participants [517] recruited from a large hospital	**C** No intervention	Yes	21-item Diet Habits Questionnaire	Fat eating habits/behavior score	**ST** Significant effects on fat eating habits/behavior
			**EXP1** CT advice				ES (1-month): −0.49
							ES (2-months): −0.18
Dutton, 2008 [77]	USA	Sedentary women [280] recruited from the general population	**C** Generic HE	Yes	National Cancer Institute Screeners	Fat intake (en%)	**MT/LT** No significant effects on fat intake
			**EXP1** Self-help booklet				
			**EXP2** CT advice				
Elder, 2005 [26]	USA	Latinas [357] recruited from the general population	**C** Generic HE	Yes	Nutrition data system: 24 h dietary recall interview	% calories from fat	**ST** Significant effects on total and saturated fat intake in EXP2 compared to EXP1
Elder, 2006 [39]			**EXP1** CT advice			Total and saturated fat intake (g)	**LT** No sustained significant effects
			**EXP2** CT advice + Promotoras				
Fries, 2005 [70]	USA	Participants [754] (18–72) recruited from physician practices	**C** No intervention	?	Fat and fiber behavior-related questionnaire	Score from 0–3	**ST** Significant effect on dietary fat behavior
			**EXP1** CT advice				ES: −0.41
							**MT** Significant effect on dietary fat behavior
							ES: −0.29
							**LT** Significant effect on dietary fat behavior
							ES: −0.23
Gans, 2009 [75]	USA	Participants [1841] with low income, recruited from waiting rooms of public health clinics	**C** Generic HE	Yes	Adapted Food Habits Questionnaire	Fat intake (Food Habits Questionnaire score: low score = high prevalence fat-lowering behavior, thus lower fat intake)	**MT** Significant effect on fat intake in EXP2 and EXP3 compared to C
			**EXP1** CT advice (at once)				ES (EXP2-C): −0.31
			**EXP2** CT advice (in 4 installments)				ES (EXP3-C): −0.31
			**EXP3** EXP2 with retailoring				
Jacobs, 2004 [61]	USA	Women [511] (50–64) recruited from nutrition and PA program (WISEWOMAN)	**C** Generic HE	Yes	54-item Dietary risk assessment	Score from 54-item scale: 0–108 not very atherogenic (0) to very atherogenic diet (108)	**LT** No significant effect on saturated fat and cholesterol intake
			**EXP1** CT advice				
Kroeze, 2008 [72]	The Netherlands	Participants [442] (18–65) recruited from companies and general population	**C** Generic HE	Yes	104-item FFQ	Total fat intake (g/day, en%)	**ST** Significant effects on total fat and saturated fat intake in EXP1 compared to C
			**EXP1** CT advice (interactive CD-ROM)			Saturated fat intake (g/day, %en)	ES (total fat): −0.31
			**EXP2** CT advice (print)				ES (saturated fat): −0.22
							**ST** Significant effects on total fat intake among risk consumers in EXP1 compared to C
							ES: −0.41
							**ST** Significant effects on total fat in EXP2 compared to C
							ES: −0.23
							**ST** Significant effects on total fat and saturated fat intake among risk consumers in EXP2 compared to C
							ES (total fat): −0.49
							ES (saturated fat): −0.42
							**MT** Significant effect on total fat and saturated fat intake among risk consumers in EXP2 compared to C
							ES (total fat): −0.53
							ES (saturated fat): −0.54
Kroeze, 2008 [73]	The Netherlands	Participants [574] (18–65) recruited from large companies and the general population	**C** Generic HE	Yes	104-item FFQ	Total fat intake (g/day)	**ST** Significant effect on awareness of fat intake in EXP1 and EXP3 compared to C
			**EXP1** CT advice (personal)		1-item	Saturated fat intake (g/day)	ES (EXP1): 0.30
			**EXP2** CT advice (personal–normative)			Self-rated fat intake (awareness) (−2 to +2)	ES (EXP3): 0.41
			**EXP3** CT advice (personal–normative–action)				**ST** Significant effect on fat intake and saturated fat intake in EXP3 compared to C
							ES (fat intake): −0.52
							ES (saturated fat intake): −0.46
							**MT** Significant effect on fat intake in EXP1, EXP2 and EXP3 compared to C
							ES (EXP1): 0.34
							ES (EXP2): 0.55
							ES (EXP3): 0.53
							**MT** Significant effect on saturated fat intake in EXP3 compared to C
							ES: −0.51
							**MT** Significant effect on fat and saturated fat intake among underestimators in EXP3 compared to C
							ES (fat intake): −0.64
							ES (saturated fat intake): -0.63
Ni Mhurchu, 2010 [53]	New Zealand	Participants [1,104] recruited from a selection of customers registered to use the Shop ‘N Go System and in-store and community-based recruitment	**C** No intervention	?	Electronic scanner (Shop ‘N Go system)	% of energy from saturated fats in purchases	**MT** No significant effect on saturated fat purchases
			**EXP1** CT advice **EXP2** CT advice + discount				
			**EXP3** Discount				
Oenema, 2008 [60]	The Netherlands	Participants [2,159] (>30) recruited from online research panel	**C** No intervention	Yes	35-item FFQ	Saturated fat intake (fat points/day from 0 to 80)	**ST** Significant effect on saturated fat intake
			**EXP1** CT advice		1-item	Self-rated intake (scale from −2 to +2)	ES: −0.16
							**ST** Significant effect on saturated fat intake in at-risk group (those who did not comply with the recommended level of saturated fat intake at baseline)
							ES: −0.23
Prochaska, 2005 [30]	USA	Sedentary primary care patients [5,407] at risk for at least one of the target behaviors recruited from primary care practices (Prochaska, 2005-458).	**C** No intervention				
Prochaska, 2004 [29]		Parents of teenagers [2,460] at risk for at least one of the target behaviors recruited from schools (Prochaska, 2005-486)	**EXP1** CT advice	Yes	22-item Dietary Behavior Questionnaire	Score on subscales: avoidance substitution modification	*Among sedentary primary care patients*
							**LT (12 months)** Significant effects on avoidance, modification and substitution
							ES (avoidance):0.24
							ES (modification):0.18
							ES (substitution):0.22
							**LT (24 months)** Significant effects on avoidance
							ES (avoidance):0.27
							ES (substitution):0.20
							*Among parents of teenagers*
							**LT (12 months)** Significant effects on avoidance and substitution
							ES (avoidance): 0.16
							ES (substitution): 0.19
							**LT (24 months)** Significant effects on avoidance and substitution
							ES (avoidance): 0.18
							ES (substitution): 0.23
Smeets, 2007 [33]	The Netherlands	Participants [2,827] (18–65) recruited from companies and the general population	**C** Generic HE	Yes	FFQ	Fat intake (g)	**MT** Significant effect on fat intake in EXP1 compared to C
De Vries, 2008 [32]			**EXP1** CT advice (once delivered in 3 months (Smeets, 2007)			Saturated fat intake (g)	ES: −0.12
			**EXP2** CT advice (3 times delivered in 9 months (De Vries, 2008)			% compliant to guidelines for saturated fat intake	**LT** Significant effect on % compliant to guideline on saturated fat intake in EXP2 compared to C
							ES: −0.18
Sternfeld, 2009 [36]	USA	Participants [787] recruited from administration offices of a large healthcare organization	**C** No intervention	Yes	Diet questionnaire based on Block Food Questionnaire	Saturated fats (g/day)	**ST** Significant effect on saturated and trans fat intake
			**EXP1** CT advice			Trans fats (g/day)	**ST** Significant effect on saturated and trans fat intake among those who chose the fats/sugar path of the intervention
							**MT** Significant effect on saturated and trans fat intake
							ES: N/A
De Bourdeaudhuij, 2007 [74]	Belgium	Participants [539] recruited from companies	**C** No intervention	Yes	48-item FFQ	Total fat intake (g/day)	**MT** Significant effect on energy from fat and total fat intake in EXP1 compared to C1 and C2
			**EXP1** CT advice on PA and fat intake sequentially delivery				Energy from fat (%)
			**EXP2** CT advice on PA and fat intake simultaneously delivered				Fat intake (seperate food groups) (g/day)
			**EXP3** CT advice only on fat intake				*EXP1 compared to C1*
							ES (energy from fat): −0.37
							ES (total fat intake): −0.32
							*EXP1 compared to C2*
							ES (energy from fat): −0.13
							ES (total fat intake): 0.09
							**MT** Significant difference in energy from fat between C1 and C2
							ES: −0.24
							**MT** Significant effect on energy from fat and total fat intake among participants who meet/do not meet fat intake recommendations in EXP1 compared to C1 and C2
							ES: N/A
Walker, 2009 [24]	USA	Women [225] (50–69) recruited from the general population	**C** Generic HE	Yes	Web-based Block98 FFQ	% calories from fat	**LT (6 months)** Significant effect on % calories from saturated fat
Walker, 2010 [25]			**EXP1** CT advice			% calories from saturated fat	ES: −0.30
							**LT (12 months)** Significant effect on % calories from saturated fat
							ES: −0.49
							**LT (18 months)** Significant effect on % calories from saturated fat
							ES: −0.56
Werkman, 2010 [56]	The Netherlands	Recent retirees [415] (55–65) recruited from pre-retirement workshops	**C** Generic HE				
			**EXP1** CT advice	Yes	Semi quantitative	Fat intake (en%)	**LT** No significant effects on fat intake
					FFQ		
Winett, 2007 [34]	USA	Participants [1,071] recruited from churches	**C** No intervention	Yes	Block98 FFQ	% kcal from fat	**LT** No significant effects on fat intake
			**EXP1** CT advice		Food shopping receipts		
			**EXP2** CT advice + church support				
C. Fruit and vegetable consumption							
Alexander, 2010 [80]	USA	Participants [2,540] (21–65) recruited from health plans	**C** Generic HE	Yes	16-item FFQ by National Cancer Institute	Fruit and vegetables intake (servings in past month)	**LT** Significant effect on fruit and vegetables intake in the past month in EXP2 compared to C
			**EXP1** CT advice		2-item	Fruit and vegetables intake (servings on a typical day)	ES: 0.10
			**EXP2** CT advice + personal counseling				**LT** Significant effect on fruit and vegetables intake on a typical day in EXP1 and EXP2 compared to C
							ES (EXP1): 0.08
							ES (EXP2): 0.13
Blair Irvine, 2004 [71]	USA	Participants [517] recruited from a large hospital	**C** No intervention	Yes	5-A-Day Screener	Fruit and vegetables consumption score	**ST** Significant effects on fruit and vegetables consumption
			**EXP1** CT advice				ES (1 month): 0.21
							ES (2 months): 0.04
Dutton, 2008 [71]	USA	Sedentary women [280] recruited from the general population	**C** Generic HE	Yes	National Cancer Institute Screeners	Fruit and vegetables intake (daily servings)	**MT/LT** No significant effects on fruit and vegetables intake
			**EXP1** Self-help booklet				
			**EXP2** CT advice				
Gans, 2009 [75]	USA	Participants [1,841] with low income, recruited from waiting rooms of public health clinics	**C** Generic HE	?	7-item National Cancer Institute fruit and vegetables screener assessment tool	Fruit and vegetables intake (servings/day)	**MT** Significant effect on fruit and vegetables intake in EXP1 and EXP2 compared to C and EXP3
			**EXP1** CT advice (at once)				ES (EXP1-C): 0.18
			**EXP2** CT advice (in 4 installments)				ES (EXP1-EXP3): 0.20
			**EXP3** EXP2 with retailoring				ES (EXP2-C): 0.12
							ES (EXP2-EXP3): 0.14
							**LT** Significant effect on fruit and vegetables intake in EXP2 compared to C
							ES: 0.17
Heimendinger, 2005 [81]	USA	Participants [3.402] (18+) recruited through Cancer Information Service offices (callers)	**C** Generic HE (1 booklet)	Yes	1-item	Fruit and vegetables intake (daily servings)	**LT** Significant effect on fruit and vegetables intake in EXP2 and EXP3 compared to C
		**EXP1** CT advice (1 booklet)			7-item FFQ		ES: N/A
		**EXP2** CT advice (4 booklets)					
		**EXP3** CT advice (4 booklets + retailoring)					
Kreuter, 2005 [79]	USA	Lower-income African–American women [1,227] (18–65) from 10 urban public health centers	**C** No intervention	Yes	13-item FFQ	Fruit and vegetables intake (servings/day)	**MT** No significant effects on fruit and vegetables intake
			**EXP1** CT advice tailored on behavioral constructs				**LT** Significant effect on fruit and vegetables intake in EXP3 compared to other groups
			**EXP2** CT advice tailored on cultural factors				**LT** Significant effect among lower motivated women on fruit and vegetables intake in EXP3 compared to other groups
			**EXP3** EXP1 + EXP2				ES: N/A
Nitzke, 2007 [78]	USA	Participants [2,024] (18–24) recruited from non-college venues	**C** No intervention	Yes	5 A Day Screener	Fruit and vegetables intake (servings)	**MT** Significant effects on fruit and fruit and vegetables intake and perceived vegetables intake ES (fruit intake): 0.12
Do, 2008 [31]			**EXP1** CT advice		2-item	Perceived daily intake	ES (fruit and vegetables intake): 0.14
					26-item FFQ	Variety in fruit and vegetables intake (number of different items consumed at least once a month, regardless of amount)	ES (perceived vegetables intake): 0.08
							**LT** Significant effects on fruit and fruit and vegetables intake and perceived intake of vegetables and fruit and vegetables
							ES (fruit intake): 0.15
							ES (fruit and vegetables intake): 0.13
							ES (perceived vegetables intake): 0.11
							ES (perceived intake fruit and vegetables): 0.12
							**LT** Significant effects on variety in fruit and vegetables consumption, consumption of seasonal fruits, juices and high beta-carotene vegetables
							ES (variety fruit) >1.00
							ES (variety vegetables) >1.00
							ES (seasonal fruits consumption) >1.00
							ES (juices consumption) >1.00
							ES (high beta-carotene vegetables consumption) > 1.00
Prochaska, 2005 [30]	USA	Sedentary primary care patients [5,407] at risk for at least one of the target behaviors recruited from primary care practices	**C** No intervention	Yes	22-item Dietary Behavior Questionnaire	Score on subscale fruit and vegetables	**LT** No significant effect on fruit and vegetables in both study samples
Prochaska, 2004 [29]		Parents of teenagers [2,460] at risk for at least one of the target behaviors recruited from schools	**EXP1** CT advice				
Smeets, 2007 [33]	The Netherlands	Participants [2,827] (18–65) recruited from companies and the general population	**C** Generic HE	Yes	FFQ	Fruit intake (pieces/day)	**MT** Significant effect on fruit intake among participants who did not meet recommendations for any behavior in EXP1 compared to C
De Vries, 2008 [32]		**EXP1** CT advice (once delivered in 3 months (Smeets et al.))				Vegetables intake (g/day)	ES: 0.30
		**EXP2** CT advice (3 times delivered in 9 months (De Vries et al.))				% compliant to guidelines for fruit intake (at least 2 pieces of fruit for 7 days/week)	**MT** Significant effect on vegetables intake in EXP1 compared to C
						Vegetables intake	ES: 0.10
						% compliant to guidelines for vegetables intake (at least 200 g of vegetables/day for 7 days/week)	**LT** Significant effect on fruit intake and % compliant to fruit guidelines in EXP2 compared to C
							ES: 0.35
							ES: 0.24
							**LT** Significant effect on vegetable intake and % compliant to vegetables guidelines in EXP2 compared to C
							ES: 0.32
							ES: 0.08
Sternfeld, 2009 [36]	USA	Participants [787] recruited from administration offices of a large healthcare organization	**C** No intervention	Yes	Diet questionnaire based on Block Food Questionnaire	Fruit and vegetables intake (cup-equivalents/day)	**ST** Significant effect on fruit and vegetables intake
			**EXP1** CT advice				**ST** Significant effect on fruit and vegetables intake among those who chose the fruit and vegetables path of the intervention
							**MT** Significant effect on fruit and vegetables intake
							ES: N/A
Van Keulen, 2011 [65]	The Netherlands	Participants [1,629] (45–70) recruited from general practices	**C1** No intervention	Yes	16-item short questionnaire	Fruit intake (servings/day)	**MT** Significant effect on fruit intake of EXP1 compared to C1 and C3
			**C2** Coaching			Vegetables (g/day)	ES (EXP1-C1): 0.19
			**C3** C2 + EXP1				ES (EXP1-C3): 0.18
			**EXP1** TC advice				**MT** Significant effect on vegetables intake of EXP1 compared to C1 and C3
							ES (EXP1-C1): 0.10
							ES (EXP1-C3): 0.12
							**LT** (~11 months) Significant effect on fruit intake of EXP1 compared to C1
							ES: 0.32
							**LT** (~11 months) Significant effect on vegetables intake of EXP1 compared to C1, C2 and C3
							ES (EXP1-C1): 0.33
							ES (EXP1-C2): 0.24
							ES (EXP1-C3): 0.19
							**LT** (~18 months) Significant effect on fruit intake of EXP1 compared to C1, C2 and C3
							ES (EXP1-C1): 0.35
							ES (EXP1-C2): 0.22
							ES (EXP1-C3): 0.24
							**LT** (~18 months) Significant effect on vegetables intake of EXP1 compared to C1
							ES: 0.27
Walker, 2009 [24]	USA	Women [225] (50–69) recruited from the general population	**C** Generic HE	Yes	Web-based Block98 FFQ	Fruit and vegetables intake (daily servings)	**LT** (6 months) Significant effect on fruit and vegetables intake
Walker, 2010 [25]			**EXP1** CT advice				ES: 0.22
							**LT** (12 months) Significant effect on fruit and vegetables intake
							ES: 0.41
							**LT** (18 months) Significant effect on fruit and vegetables intake
							ES: 0.40
Werkman, 2010 [56]	The Netherlands	Recent retirees [415] (55–65) recruited from pre-retirement workshops	**C** Generic HE	Yes	Semi quantitative	Fruit and vegetables intake (g/MJ)	**LT** No significant effect on fruit and vegetables intake
			**EXP1** CT advice		FFQ		
Winett, 2007 [34]	USA	Participants [1,071] recruited from churches	**C** No intervention	Yes	Block98 FFQ	Fruit and vegetables intake (g/1000 kcal)	**LT (7 months)** Significant effect on fruit and vegetables intake in EXP1 compared to C
			**EXP1** CT advice		Food shopping receipts		ES: 0.44
			**EXP2** CT advice + church support				Significant effect on fruit and vegetables intake in EXP2 compared to C
							ES: 0.57
							**LT (16 months)** Significant effect on fruit and vegetables intake in EXP1 compared to C
							ES: 0.12
							Significant effect on fruit and vegetables intake in EXP2 compared to C
							ES: 0.32
D. Other dietary topics							
Adachi, 2007 [28]	Japan	Overweight Japanese women [205] recruited from the general population (Adachi, 2007)	**C1** Self-help booklet	?	Weight parameters	BMI (kg/m^2^)	**ST** Significant effect on BMI in EXP1 & EXP2 compared to C1 & C2 among overweigh Japanese women
Tanaka, 2010 [27]		Overweight Japanese men [51] recruited from the general population (Tanaka, 2010)	**C2** C + self- monitoring of weight and walking				*BMI*
			**EXP1** CT advice				ES EXP1-C1: −0.60
			**EXP2** ^f^ CT advice + self- monitoring of weight and walking				ES EXP1-C2: −0.48
							ES EXP2-C1: −0.77
							ES EXP2-C2: −0.66
							**ST** Significant effect on BMI in EXP2 compared to C1among overweigh Japanese *men*
							*BMI*
							ES EXP2-C1: −0.69
							**MT** Significant effect on BMI in EXP2 compared to C1 & C2 among overweight Japanese women
							BMI
							ES EXP2-C1: −0.70
							ES EXP2-C2: −0.58
							**LT** Significant effect on BMI in EXP2 compared to C1 and C2 among overweight Japanese women
							*BMI*
							ES EXP2-C1: −0.59
							ES EXP2-C2: −0.55
							**LT** No significant effect on BMI in EXP2 compared to C1among overweigh Japanese men
Elder, 2005 [26]	USA	Latinas [357] recruited from the general population	**C** Generic HE	Yes	Nutrition data system (NDS): 24 h dietary recall interview	Total energy intake (kcal)	**ST/LT** No significant effects
Elder, 2006 [39]			**EXP1** CT advice			Total carbohydrates intake (g)	
			**EXP2** CT advice + promotoras				
Fries, 2005 [70]	USA	Participants [754] (18–72) recruited from physician practices	**C** No intervention	?	Fat and fiber behavior-related questionnaire	Score from 0–3	**ST** Significant effect on fiber behavior
			**EXP1** CT advice				ES: −0.35
							**MT** Significant effect on fiber behavior
							ES: −0.24
Haapala 2009 [55]	Finland	Overweight participants [125] (25-44) from the general population	**C** Generic HE	Weight parameters	Body weight (kg)		**LT** Significant effect on weight loss and waist circumference
			**EXP1** CT advice		% Weight loss		ES (weight loss): −0.14
					Waist circumference		ES (waist circumference): −0.18
Kroeze, 2008 [72]	The Netherlands	Participants [442] (18–65) recruited from companies and general population	**C** Generic HE	Yes	104-item FFQ	Energy intake (MJ/day)	**ST** Significant effects on energy intake in EXP1 and EXP2 compared to C
			**EXP1** CT advice (CD-ROM)				ES: −0.28
			**EXP2** CT advice (print)				ES: −0.38
							**ST** Significant effects on energy intake among risk consumers in EXP1 and EXP2 compared to C
							ES: −0.50
							ES: −0.66
							**MT** Significant effects on energy intake among risk consumers in EXP1 and EXP2 compared to C
							ES: −0.68
							ES: −0.44
							**MT** Significant effects on energy intake in EXP2 compared to C
							ES: −0.26
Poddar, 2010 [82]	USA	College students [294] recruited from a land grant, research-intensive university	**C** No intervention	?	7 day food records	Average daily dairy servings	**MT** No significant effect
			**EXP1** CT advice				
Prochaska, 2008 [54]	USA	Participants [1400] at risk for at least one risk behavior (exercise, stress, BMI >25 kg/m^2^ and smoking) recruited from a major medical university	**C** Health Risk Assesment	Yes	Self-report	% above/below BMI = 25 kg/m^2^	**MT** No significant effect on BMI
			**EXP1** C + coaching				
			**EXP2** C + TTM-based feedback				
Rothert, 2006 [38]	USA	Overweight and obese (BMI = 27–40 kg/m^2^) participants [2862] recruited from health care delivery system	**C** Generic HE	?	Self-report	% of baseline weight lost	**MT/LT** Significant effect on % of baseline weight lost
			**EXP1** CT advice				ES > 1.00
Sternfeld, 2009 [36]	USA	Participants [787] recruited from administration offices of a large healthcare organization	**C** No intervention	Yes	Diet questionnaire based on Block Food Questionnaire	Added sugars (g/day)	**ST/MT** No significant effects on added sugars
			**EXP1** CT advice				
Walker, 2009 [24]	USA	Women [225] (50–69) recruited from the general population	**C** Generic HE	Yes	Web-based Block98 FFQ	Whole-grain intake (daily servings)	**LT** No significant effects
			**EXP1** CT advice		Bioelectrical impedance analysis	% Body fat	
					Weight parameters	BMI (kg/m^2^)	
Werkman, 2010 [56]	The Netherlands	Recent retirees [415] (55–65) recruited from pre-retirement workshops	**C** Generic HE	Yes	Weight parameters	Waist circumference (cm), BMI (kg/m^2^)	**LT** Significant effect on waist circumference among men with low education
			**EXP1** CT advice		Semi quantitative	Energy intake (MJ/day)	
					FFQ		
Winett, 2007 [34]	USA	Participants [1,071] recruited from churches	**C** No intervention	Yes	Block98 FFQ	Fiber intake (g/1,000 kcal)	**LT (7 months)**
			**EXP1** CT advice		Weight parameters	Weight (lb)	Significant effect on fruit and vegetables intake in EXP1 compared to C
			**EXP2** CT advice + church support		Food shopping receipts		*ES:* 0.35
							Significant effect on fruit and vegetables intake in EXP2 compared to C
							*ES:* 0.44
							Significant effect on weight
							In EXP2 compared to C
							*ES: 0.21*
							**LT (16 months)**
							Significant effect on fruit and vegetables intake in EXP1 compared to C
							*ES:* 0.20
							Significant effect on fruit and vegetables intake in EXP2 compared to C
							*ES:* 0.28

*C* control condition, *EXP1* experimental condition 1, *EXP2* experimental condition 2, *EXP3* experimental condition 3, *ES* effect size, *[125]* 125 participants, *(50–69)* 50 to 69 years old, *HE* health education, *(L/M/V/MV) PA* (low-/moderate-/vigorous-/moderate to vigorous-intensity) physical activity, *CT* computer-tailored, *VO2max* maximal oxygen uptake, *MET* metabolic equivalent, *FFQ* food frequency questionnaire, *IPAQ* International Physical Activity Questionnaire, *SQUASH* Short Questionnaire Assessing Health-enhancing physical activity, *AQuAA* Activity Questionnaire for Adolescents and Adults, *BMI* body mass index, *N/A* not available

^a^Some publications reported on the characteristics and effects of the same intervention and are therefore clustered in one cell

^b^No intervention equals no info in the 2006 review; generic HE equals generic info in the 2006 review

^c^Significant effect = effect that reached statistical significance (*p*<0.05)

^d^Effect sizes were calculated when mean and SD were available at post-test and a significant effect in favor of tailoring had been found. ES is interpreted according to Cohen’s guidelines [67] based on an application in Dolan et al. [69]; cutoff values of 0.2–0.5 = small, 0.5–0.8 = moderate, and >0.8 = large effects

^e^Short term (ST), <3 months; medium term (MT), 3–6 months; long term (LT), >6 months

^f^In the study of Tanaka et al. [27], only EXP2 versus the self-help booklet was tested
