# Supplementary material for: A Systematic Review of Randomized Controlled Trials on the Effectiveness of Computer-Tailored Physical Activity and Dietary Behavior Promotion Programs: an Update
Source: Ann Behav Med. 2012 Jul 6;44(2):259–86. doi: 10.1007/s12160-012-9384-3 (PMC3442159; doi:10.1007/s12160-012-9384-3)
Supplement: Supplementary file 1 — (DOCX 53 kb) [file 12160_2012_9384_MOESM1_ESM.docx]

Electronic Supplementary Material**:** Intervention characteristics

| **1st author(s)**  **[reference number]** | **Target behavior(s)** | **Theories** | **Tools** | **Tailoring variables** | **Feedback frequency** | **Additional strategies/notes** |
| --- | --- | --- | --- | --- | --- | --- |
| **A: PHYSICAL ACTIVITY** | | | | | | |
| Adachi, 2007 [30]  Tanaka, 2010 [29] | Exercise | Behavioral therapy | Print | Personal characteristics  Physical activity  Readiness to change behaviors  Weight history  Weight rebound experience  Primary purpose of weight control  Target weight  Body image | 2 | Intervention primary focuses on weight control and secondary on exercise and dietary habits  Booklet of behavioural weight control |
| Caroll, 2010 [99] | Physical activity |  | Email reports | Physical activity  Stage of change  Processes of change  Self-efficacy  Barriers  Benefits | 4 |  |
| Dunton, 2008 [68] | Physical activity | TTM  HBM | On screen | Physical activity  Stage of change  Barriers  Motivators | 1 | 10 weekly newsletters supporting the tailored advice & encouraging further learning |
| Hageman, 2005 [72] | Physical activity | HPM | On screen newsletters | Physical activity  Benefits  Barriers  Self-efficacy  Goals | 3 |  |
| Hurling, 2007 [42] | Physical Activity | Social comparison  ELM  Goal setting  Decisional Balance Theory | On screen | Physical activity  Barriers  Solutions  Goal setting | 1 | Email and/or telephone reminders  Online schedule to plan weekly exercise sessions  Message board |
| Jacobs, 2004 [100] | Physical activity | SCT  TTM  RPT | Print/telephone | Behavioral goals  Stage of change  Knowledge  Social Support  High risk situations for relapse  Benefits  Barriers | 8 | Intervention also focuses on dietary intake |
| Marcus, 2007 [73] | Physical activity | TTM  SCT | Telephone/print | Physical activity  Stage of change  Processes of change  Decisional balance | 14 | Stage-targeted booklets  Tip sheets |
| Marcus, 2007 [74] | Physical activity | TTM  SCT | On screen/print | Physical activity  Stage of change  Processes of change  Decisional balance | 16 | Educational materials and tips |
| Napolitano, 2006 [35]  Dutton, 2008^2^ [36] | Physical activity | TTM  SCT | Print | Stage of change  Processes of change  Self-efficacy  Decisional balance | 4 |  |
| Oenema, 2008 [66] | Physical activity | PAPM | On screen and/or print | (Perceived) Physical activity  Awareness  Stage of change  Attitude  Self-efficacy  Implementation intentions  Demographics | At least 1 (more visits possible) | Intervention also focuses on dietary intake and smoking |
| Pekmezi, 2009 [101] | Physical activity | TTM  SCT | Print | Stage of change  Processes of change  Self-efficacy  Motivational readiness | 6 | Physical activity logs with tip sheets |
| Prochaska, 2008 [60] | Exercise | TTM | On screen | Stage of change  Self-efficacy  Processes of change  Benefits  Barriers | 3 (recommended) | Intervention also focuses on smoking, stress dietary intake |
| Quintiliani, 2010 [65] | Physical activity | TTM  HBM  Social learning theory | On screen | Physical activity  Stage of change  Perceived barriers | 1 |  |
| Rothert, 2006 [43] | Physical activity |  | On screen | Physical activity  Demographics  Experiences on weight loss  Personal/family health history  Attitude  Barriers  Social support  Goals  Expectations  Preferences  Self-efficacy | 4 | Intervention primary focuses on weight control  Encouraging email messages from buddy |
| Slootmaker, 2009 [40] | Physical activity |  | On screen | Physical activity  Preferences  Barriers | 1 (more visits optional) |  |
| Smeets, 2007 [38]  De Vries, 2008 [37] | Physical activity | I-Change model | Print | Physical activity  Stage of change  Awareness  Motivation  Attitude  Self-efficacy | 1-3 | Intervention also focuses on smoking and fruit and vegetables and fat intake  Smeets at al evaluated the first computer-tailored letter at short-term, De Vries et al evaluated the effects of three letters with an action planning component randomly applied in 3th letter |
| Smeets, 2008 [70] | Physical activity | I-Change model | Print | Physical activity  Stage of change  Social support  Preferences  Benefits  Barriers | 1 |  |
| Spittaels, 2007 [69] | Physical Activity | TPB  TTM | On screen | Physical activity  Social support  Intention  Stage of change  Knowledge  Self-efficacy  Attitude  Barriers  Benefits | 1/2 | Non-tailored emails with invitation to revisit website |
| Spittaels, 2007 [104] | Physical Activity | TPB  TTM | On screen | Physical activity  Stages of change  Social support  Intention knowledge  Attitude  Self-efficacy  Barriers  Benefits | 1 | Stage of change targeted email tip sheets |
| Sternfeld, 2009 [41] | Physical activity |  | Email reports | Physical activity  Stage of change  Self-efficacy  Individual lifestyle constraints  Physical activity preferences | 12 | Intervention also focuses on saturated and trans fats intake, fruit and vegetables intake  Personal homepage: tips on how to achieve goals  Weekly health note  Simulation tools  Progress tracking tool  Review of barriers  Discussion board  Links to additional resources  Reminder messages |
| Van Keulen, 2011 [71] | Physical activity | I-Change model  Control Theory | Print | Physical activity  Awareness  Demographics  Stage of change  Attitude  Self-efficacy  Expectations  Action plans | 4 |  |
| Van Stralen, 2009 [23]  Van Stralen, 2011 [24] | Physical activity | I-Change model  TTM  HPA  PAPM  SRT  SDT | Print | Basic tailored intervention:  *Letter 1:*  (self-estimated) Physical activity  Stage of change  Age  Gender  Attitude  Self-efficacy  Benefits  Social support  *Letter 2:*  Attitude  Self-efficacy  Possibilities  Social support  *Letter 3:*  Changes in physical activity and determinants  Intervention Plus  Additional: location | 3 | Access to forum and e-buddy system (Intervention Plus) |
| Walker, 2009 [25]  Walker, 2010 [26] | Physical activity | HPM | Email newsletter | Benefits  Barriers  Self-efficacy  Habits  Family Support | 18 | Instructional videotapes |
| Wanner, 2009 [67] | Physical activity | TTM | On screen | Physical activity  Stage of change  Decisional balance  Processes of change  Self-efficacy  Attitude  Knowledge | 1 (3 invitations for re-visit) | Strength and stretching exercise sheets  organizational and motivational download forms |
| Werkman, 2010 [62] | Physical activity | Intervention mapping protocol | CD-ROM/print | CD-ROM I (module 2)  BMI  BMI-related health consequences Energy-balance behavior  CD-ROM II (module 3)  Physical activity  Letter (module 5)  Physical activity | 3 | Intervention (5 modules) also focuses on fat, fruit and vegetables intake and weight loss  Encouraging/informative newsletters |
| Winett, 2007 [39] | Physical activity |  | On screen | Daily step counts  Goal attainment  Strategies  Preferred reasons for using intervention (health/weight loss) | 12 | Intervention also focuses on fruit and vegetables intake, fat intake, fiber intake and weight loss  Church-based supports |
| **B. DIET** | | | | | | |
| Adachi, 2007 [30]  Tanaka, 2010 [29] | Dietary habits | Behavioral therapy | Print | Personal characteristics  Readiness to change behavior  Weight history  Weight rebound experience  Primary purpose of weight control  Target weight  Body image  Eating habits | 2 | Intervention primary focuses on weight control and secondary on exercise and dietary habits  Booklet of behavioural weight control |
| Alexander, 2010 [82] | Fruit and vegetables intake | SCT  TTM  HBM | On screen | Needs  Dietary preferences, Interests | 4 | Intervention also included optional short video/audio files of behavioral strategies and/or recipe preperations |
| Blair Irvine, 2004 [76] | Fat intake, Fruit and vegetables intake | TTM  TRA  SCT  HCT | On screen | Stage of change  Attitude  Intentions  Self-efficacy  Demographics  Eating habits  Environmental factors | 1 (more visits optional) | Intervention also includes interactive multimedia combining audio, video, graphics and printout |
| Elder, 2005 [44]  Elder, 2006 [45] | Calories from fat, Fiber intake, Energy intake, Total and saturated fat intake, Carbohydrates intake | Lay Health Advisor Model | Print | BMI  Top 10 meals prepared at home  Readiness to change  Points of influence for change | 12 | Intervention also includes 12 weekly home visits and activity inserts in newsletters |
| Fries, 2005 [75] | Fat behavior, Fiber behavior | SCT  TTM  SMM | Email report | Fat behavior  Fiber behavior | 1 | Counselling phone call and self-help booklets |
| Gans 2009 [80] | Fat intake, Fruit and vegetables intake | TTM  SCT | Print | Fruit and vegetables intake, Fat-related behavior  Demographics  Self-efficacy  Barriers  Interests | 1- 4 | Intervention also includes motivational DVD |
| Haapala,2009 [61] | Weight loss |  | On screen (mobile phone text messages) | Weight  Daily energy requirement | on demand |  |
| Heimendinger, 2005 [83] | Fruit and vegetables intake | TTM  HBM  SCT | Print | Fruit and vegetables intake  Stage of change  Outcome expectations  Barriers  Benefits  Skills  Environmental factors | 1/2 |  |
| Jacobs, 2004 [67] | Saturated fat intake, Cholesterol intake | SCT  TTM  RPT | Print/telephone | Behavioral goals  Stage of change  Knowledge  Social Support  High risk situations for relapse  Benefits  Barriers | 8 |  |
| Kreuter, 2005 [81] | Fruit and vegetable intake |  | Print magazines | Demographics  EXP1  Fruit and vegetables intake  Knowledge  Beliefs  Perceived Barriers  Stage of readiness  Self-efficacy  Exposure to and preference for different fruit and vegetables  Having received a recommendation  Interest in eating more fruit and vegetables  Perceived importance  Environmental factors  EXP2  Religiosity  Collectivism  Racial pride  Time orientation | 6 | Intervention also included promotion of mammography use (participants aged 40-65) |
| Kroeze, 2008 [77] | Fat intake | PAPM  TPB | On screen/print | Perception of own fat intake (high-low)  Attitude  Self-efficacy  Readiness to change  Environmental factors  Demographics | 1 |  |
| Kroeze, 2008 [78] | Fat intake | PAPM  TPB | Print | EXP1 + EXP2  Fat intake  EXP3  Fat intake  Self-efficacy  Intention to change  Attitude | 1 |  |
| Mhurchu, 2010 [59] | Saturated fat purchases |  | Print | Usual food purchases | 6 |  |
| Nitzke, 2007 [34]  Do, 2008 [33] | Fruit and vegetable intake | TTM | Print | Fruit and vegetables intake  Decisional balance  Stage of change  Processes  Self-efficacy | 6 | Magazines  2 boostercalls at 4 wks and 4 months post-baseline |
| Oenema, 2008 [66] | Saturated fat intake | PAPM | On screen and/or print | (perceived) Intake of saturated fats  Awareness  Stage of change  Attitude  Self-efficacy  Implementation intentions  Demographics | at least 1 (more visits possible) | Intervention also focuses on physical activity and smoking |
| Poddar, 2010 [84] | Dairy intake | SCT | On screen | Dairy intake | 23 |  |
| Prochaska, 2005 [32]  Prochaska, 2004 [31] | Fat intake, Fruit and vegetables intake |  | Print | Stage of change  Readiness to change  Decisional balance  Change processes  Self-efficacy | 3 | Intervention also focuses on smoking, skin cancer prevention regular mammography use  Integrated multiple risk behavior stage-matched self-help manual |
| Rothert, 2006 [43] | Dietary behavior |  | On screen | Dietary behavior  Demographics  Experiences on weight loss  Personal/family health history  Attitude  Barriers  Social support  Goals  Expectations  Preferences  Self-efficacy | 4 | Intervention primary focuses on weight control  Encouraging email messages from buddy |
| Smeets, 2007 [38]  De Vries, 2008 [37] | Fat intake, Fruit and vegetables intake | I-Change model | Print | Fruit and vegetables intake  Fat intake  Awareness  Motivation  Stage of change  Attitude  Self-efficacy | 1-3 | Intervention also focuses on smoking and physical activity Smeets at al evaluated the first computer-tailored letter at short-term, De Vries et al evaluated the effects of three letters with an action planning component randomly applied in 3th letter |
| Sternfeld, 2009 [41] | Saturated and trans fat intake, Fruit & vegetables intake, Added sugars |  | Email reports | Dietary intake  Stage of change  Self-efficacy Individual lifestyle constraints | 12 | Intervention also focuses on physical activity  Personal homepage: tips on how to achieve goals  Weekly health note  Simulation tools  Progress tracking tool  Review of barriers  Discussion board  Links to additional resources  Reminder messages |
| Van Keulen, 2011 [71] | Fruit and vegetables intake | I-Change model  Control Theory | Print | Fruit/vegetables intake  Awareness  Demographics  Stage of change  Attitude  Self-efficacy  Expectations  Action plans | 4 |  |
| de Bourdeaudhuij, 2007 [79] | Fat intake | TPB  TTM | On screen and/or print | Fat intake  Intentions  Attitude  Self-efficacy  Social support  Knowledge  Benefits  Barriers  Demographics | 1/2 |  |
| Walker, 2009 [25]  Walker, 2010 [26] | Fat intake, Fruit and vegetables intake | HPM | Print | Benefits  Barriers  Self-efficacy  Habits  Family Support | 18 | Instructional videotapes  Action planning |
| Werkman, 2010 [62] | Fat intake, Fruit and vegetables intake | Intervention mapping protocol | CD-ROM/print | CD-ROM I (module 2)  BMI  BMI-related health consequences  Energy-balance behavior  CD-ROM II (module 3)  Fibre consumption  Portion sizes of energy dense foods  Fat consumption  Letter (module 5)  Body weight  Dietary intake | 3 | Intervention (5 modules) also focuses on physical activity and weight loss. Modules 1 and 4 are not tailored  Encouraging/informative newsletters |
| Winett, 2007 [39] | Fat intake, Fruit and vegetables intake |  | On screen | Nutrition  Goal attainment  Strategies  Preferred reasons for using intervention (health/weight loss) | 12 | Intervention also focuses on fruit and vegetables intake, fat intake, fiber intake and weight loss  Church-based supports |

_TPB = Theory of Planned Behavior; TTM = Transtheoretical Model; HBM = Health Belief Model; HPA = Health Promotive action and Preventive action model; SMM = Social Marketing Model; TRA = Theory of Reasoned Action; SCT = Social Cognitive Theory; ELM = Elaboration Likelihood Model; HPM = Health Promotion Model; HCT = Health Communication Theory; PAPM = Precaution Adoption Process Model; SRT = Self-regulation Theory; SDT = Self-determination theory; RPT = Relapse Prevention Theory; EXP1 = experimental condition 1; EXP2 = experimental condition 2; EXP3 = experimental condition 3_

1. _Some publications reported on the same intervention, and are therefore clustered in one cell_
2. _Dutton, 2008 examined the effects of an intervention aimed at physical activity on dietary intake._
